# Adolescents and primary herpetic gingivostomatitis: an Italian overview

**DOI:** 10.1007/s11845-021-02621-3

**Published:** 2021-05-15

**Authors:** Elena Bardellini, Francesca Amadori, Federica Veneri, Giulio Conti, Alberto Paderno, Alessandra Majorana

**Affiliations:** 1 Department of Medical and Surgical Specialties, Radiological Sciences and Public Health, School of Paediatric Dentistry- Dental Clinic, University of Brescia, ASST-Spedali Civili of Brescia, 25133 Brescia, Italy; 2 University Vita-Salute S. Raffaele, 10090 Milan, Italy; 3 Unit of Otorhinolaryngology-Head and Neck Surgery, Department of Medical and Surgical Specialties, Radiologic Sciences, and Public Health, ASST Spedali Civili Di Brescia, University of Brescia, 25133 Brescia, Italy

**Keywords:** Adolescent, Gingivitis, Herpes, Therapy, Verbascoside

## Abstract

**Aim:**

The aim of this study was to investigate the therapies administered to Italian adolescents with primary herpetic gingivostomatitis (PHGS)

**Methods:**

The medical records of 74 adolescents with PHSG were reviewed. The following data were recorded: age, gender, day of onset, type of treatment, lesions’ severity, pain scoring, eating, and drinking ability. The oral examination was performed at the first evaluation (T0) and after one week (T1).

**Results:**

All patients showed up at the first visit at least 48 h after the onset of symptoms. No patient was prescribed an antiviral therapy. An antibiotic therapy was prescribed in order to prevent secondary bacterial infections**.** Fifteen patients had been treated with non alcoholic chlorhexidine rinses (group A), 29 patients with non alcoholic chlorhexidine rinses plus hyaluronic acid gel (group B); 30 patients with non alcoholic chlorhexidine rinses plus Mucosyte® (group C). A significant improvement of the pain scoring and lesions’ severity was noted in group C.

**Conclusion:**

In Italian adolescents, PHGS is diagnosed at least 48 h after onset and the antibiotic therapy is widely prescribed in order to prevent overinfections. Among topical therapies, an association of verbascoside and sodium hyaluronhate seems to favour a faster healing.

## Introduction

Primary herpetic gingivostomatitis (PHGS) is the most commonly observed clinical manifestation of primary herpes simplex virus (HSV 1–2) infection, occurring in 25–30% of affected children and adolescents, with two peaks in age. The first peak occurs in children between 6 months and 5 years old, and the second peak occurs in young adults in their early 20 s [1, 2].

Clinical features of PHGS include prodromal fever and symptoms, followed by oral and extra-oral lesions. Prodromal, non-pathognomonic, general signs, and symptoms are fever, chills, nausea, loss of appetite, lethargy, irritability, malaise, and headache. In children, prodromal symptoms are often the only signs of primary HSV-1 infection and sometimes they can be so mild (or even non existent) that the affected subjects cannot recognize them. Only 10–12% PHGS cases among children are so severe that parents notice them. On the contrary, PHGS in younger adults is more severe [3, 4]. The lesions begin as vesicles, which coalesce to form painful ulcers with generalized edematous and bleeding gingivae. Associated findings include fever, bad breath, refusal to drink, anorexia, and submandibular or cervical lymphadenitis. Lesions occur on the tongue, vermillion, buccal, hard and soft palatal mucosae, and, less frequently, pharyngeal and nasal mucosae [5, 6]. Moreover, the affected gingivae often exhibit discernible erosions along the mid-facial free gingival margins, and these may precede the appearance of the mucosal vesicles.

Conventional therapy suggests treating children and adolescents with antiviral therapies (acyclovir or valacyclovir or famciclovir) only within the first 72 h of symptom onset, as long as they have clear symptoms of gingivostomatitis and suffer from substantial pain or dehydration [3].

The severity and quantity of intraoral lesions may significantly reduce dietary intake and predispose the patient to dehydration. Thus, it is important to balance any decrease in intake with fluids. Either nutritional supplements or a pureed or a blended diet, is sufficient until the patient can tolerate solid food. Most systemic analgesics such as paracetamol are adequate to manage the associated pain and malaise [3]. In most cases, the treatment of lesions involves topical therapy and possible antibiotic therapy to reduce the risk of bacterial superinfections.

Since they are oral lesions, the young patients often turn to their dentist. As far as we know, there are no studies investigating the dentist’s therapeutic approach to PHGS, especially in adolescents. This study aims to investigate the therapies administered in our department (Dental Clinic of the University of Brescia, Italy) for PHGS in adolescents and to evaluate their effectiveness in terms of reduction of lesions size and pain relief.

## Material and methods

### Sample selection

All the medical records of adolescents with oral mucosal lesions (OMLs) visited in the period from January 2014 to December 2019 in our department were reviewed. The medical records of the patients with OMLs were filled out by three clinicians who had undergone the same training and therefore permitted the standardization of the procedures. The calibration to detect OMLs was the same as our team’s previous study [7].

Inclusion criteria were (a) patients aged 13–18 years old, (b) clinical evidence of PHGS (diffuse oral ulcerations, edematous gingivae, lymphadenopathy, fever, and malaise), and (c) laboratory evidence of herpes virus infection (positive sierology results and positive culture results). Exclusion criteria were (a) exposure to immunosuppressants, including inhaled cortico-steroids; (b) chronic infection, including HIV infection; (c) malignancy (solid tumours, haematological diseases…); and chemo-radiotherapy (c) bone marrow or organ transplantation.

### Study design

This study was designed as a retrospective cross-sectional study. The following data were recorded from each patient’s medical record: age at the time of diagnosis, gender, day of onset of the lesions, type of treatment (topical and/or systemic), lesions’ severity, pain, eating, and drinking ability. The oral examination was performed at the first visit (T0) and after 1 week of treatment (T1).

**Lesion severity.** The oral lesions were classified as mild (up to 10 lesions on the tongue or oral mucous membrane), moderate (11 to 20 lesions with swelling of the gums), or severe (> 20 on the tongue or oral lesions and gum lesions) [8].

**Pain Scoring.** Pain was evaluated through the Visual Analogue Scale (VAS). In this system, one indicates no pain and ten indicates severe pain; patients were asked to select a number from 1 to 10 on a ruler with drawn faces to express the intensity of their pain (Wong Baker face scale).

**Eating and drinking ability.** They were classified as normal, less than normal and unable to eat or drink.

### Data analysis

The data were inserted on a spread sheet. A 5% level of significance was used and the data was analysed using R® software for Mac. Descriptive analysis was used for demographical data. Differences in severity and eating and drinking ability were calculated by chi square test; *p* < 0.05 was considered statistically significant.

About VAS, to calculate the difference between T0 and T1 for the same group, the non-parametric Mann Whitney *U*-test was used; to investigate the difference at the end of the treatment for all groups, one-way Anova test was chosen. To underline which groups are significantly different, Turkey HSD post hoc test was used.

### Ethical considerations

This study was approved by the local Ethic Committee (NP. 4287). We conducted that this study was conducted in university hospital and no companies had any role in the design and the conduct of the study, collection, management, and analysis of the data.

## Results

Out of 1324 adolescents with OML, a total of 84 teenagers (6.3%) showed PHGS in the mentioned period. At the first visit patients showed at least 48 h after symptoms’ onset (i.e., from the 2nd to the 4th days). No patient was prescribed an antiviral therapy. An antibiotic therapy was prescribed in 74 cases in order to prevent secondary bacterial infections. Ten patients did not receive any antibiotic prescription and were treated only with non-alcoholic chlorhexidine rinses. To make the samples homogeneous in order to compare the efficacy of different treatments, we considered only the 74 patients undergoing antibiotic therapy. Fifteen patients out of all of them had been treated with non-alcoholic chlorhexidine rinses (group A), 29 patients with non-alcoholic chlorhexidine rinses plus hyaluronic acid gel (group B); 30 patients with non-alcoholic chlorhexidine rinses plus Mucosyte® (group C). All groups were homogeneous for the variables and no differences were recorded.

### Severity of the lesions

At T1, after 1 week, group C showed a significantly larger number of patients with complete healing of the oral mucosa (*p* = 0.01) or with an improvement of the *moderate* lesions (*p* = 0.0297) compared to group A. No difference between group A and group B nor between B and C was recorded for the mild or severe lesions. As to the differences within each group at T0 and T1, all therapies showed to be efficient, except for mild lesions that in all groups did not show a statistically significant improvement.

### Eating and drinking ability

At T1, all patients totally or partially improved the eating and drinking ability; a significant statistically difference was noted between group A and group C in total recovery (*p* = 0.0004) and in partial recovery (*p* = 0.0004). Group treated with Mucosyte® showed a full improvement of the abilities.

### Pain scoring

The medians of VAS and *p*-values are displayed in Table 1. A statistically significant difference was obtained from T0 to T1 for all groups (*p* < 0.005).

**Table 1:** Severity of the oral lesions at T0 and T1

	T0 Group A (*n* = 15)	T0 Group B (*n* = 29)	T0 Group C (*n* = 34)	T1 Group A (*n* = 15)	T1 Group B (*n* = 29)	T1 Group B (*n* = 34)
-No lesions	0	0	0	10	25	32
-Mild	3	6	5	3	2	2
-Moderate	8	12	18	2	2	0
-Severe	4	11	11	0	0	0

At T1, post hoc test showed that the VAS values significantly decreased in group C with respect to those in group A (*p* = 0.0012) (Table 2).

**Table 2:** Pain scoring (VAS scale and eating/drinking ability) at T0 and T1

	T0 Group A (*n* = 15)	T0 Group B (*n* = 29)	T0 Group C (*n* = 34)	T1 Group A (*n* = 15)	T1 Group B (*n* = 29)	T1 Group B (*n* = 34)
VAS	7.73 ± 0.70	7.86 ± 0.87	7.79 ± 0.84	2.86 ± 0.35	2.41 ± 0.98	2.79 ± 1.06
Eating and drinking ability						
- Normal	0	0	0	6	25	34
- Less than normal	8	15	22	5	4	0
- Unable	2	14	12	0	0	0

## Discussion

Nearly all human beings, by the time they reach adolescence, are infected with multiple herpes viruses. Typical primary oral HSV-1 infection is characterized by productive infection and lytic activity directed against the epithelial cells and limited to the site of entry. The consequent local inflammatory process, possibly enhanced by co-infection, leads to the development of the PHGS after an incubation of 2–20 days [4]. Besides gingival and mucosal ulcers, PHGS show signs, such as lymphadenopathy, oral malodour, and coated tongue, due to the accumulation of bacteria and yeasts on the tongue, which in turns is due to soreness. The latter leads to limited function of the oral muscles, with consequently reduced bacterial clearance from the oral cavity and, to minor extent, to poor oral hygiene and co-infections [4]. This is the reason for which most of the adolescents of our department resulted under antibiotic therapy. In addition, no adolescent showed up to hospital observation within 48 h from the first clinical signs, and consequently, none of them was prescribed an antiviral therapy. The most common morbidities after PHGS in adolescents are pain, oedema, and poor oral intake. Controlling pain is a challenging task in those patients. Pain is due to inflammation, nerve irritation, and pharyngeal spasm [2]. Along with acyclovir, other drugs are used for controlling pain such as non-steroidal anti-inflammatory drugs, topical anesthetizing sprays, or sucralfate. However, the efficacy and side effects of these agents necessitate more surveys to find the suitable pain relieving drugs beside acyclovir [2]. A suitable topical therapy seems to be important for the relief of symptoms.

This retrospective study was designed to investigate the type and efficacy of different topical therapies prescribed in our department in adolescents over a 6-year period. We found that an antiseptic topical therapy (non-alcoholic chlorexidine rinses 0.2% twice a day) was prescribed to all adolescents with PHGS. The fact that all adolescents have undergone antibiotic and antiseptic therapy makes clear the clinician’s intention to contain the risk of superinfections and certainly the tendency in Italy to easily prescribe antibiotic prophylaxis in all suspected cases. This is in accordance with a recent study about antibiotic in the paediatric population attending emergency departments in Lombardy, Italy [9]. Antibiotics are the most frequently prescribed drugs in the paediatric population in both national and international contexts, with an overall prevalence of 47.3%, and about half the prescriptions are unnecessary. Large qualitative and quantitative differences in the antibiotic prescription profiles for paediatric outpatients have been found between and within countries, and Italy has one of the highest prevalence of prescriptions and most frequent use of second-choice antibiotics (i.e., cephalosporin and macrolides). There may be several reasons for these differences: sociocultural factors, education and income, and physician’s attitude seem to play major roles [10]. More must be done to improve rational use of antibiotics and educational interventions including physicians in both setting are strongly needed.

According to our results, the patients treated with Mucosyte ® showed a significantly larger number of patients with complete healing of the oral mucosa or with an improvement of the moderate lesions. No difference among the groups was recorded regarding the improvement of mild or severe lesions.

Being similar the results in group B and C, the reparative action seems to be linked to the sodium hyaluronhate, which acts as a physical barrier between the oral environment and oral mucosa, inducing also biomolecular and physiological changes in keratinocytes and mesenchimal cells. Regarding the pain perception, all groups experienced a statistically significant reduction in terms of pain, from T0 to T1, but comparing the three groups it emerged a more consistent relief in group C than in group A. As for the ability to eat and drink, all patients totally or partially improved; group C was the only one reporting a statistically significant “total” recovery. These latter results can be more properly depending on the presence of verbascoside. Verbascoside is the extract of syring vulgaris, and it belongs to the fenilpropanoid family, a new class of anti-inflammatory drugs. Previous studies have suggested that verbascoside can prevent the oxidative stress since it reduces the production of superoxide radicals and consequently reduces the activity of iNOS and COX-2 [11]. Moreover, verbascoside induces the dose-dependent decrease of the expression of IL8, inhibiting proinflammatory activity of enzymes like COX-2 and iNOS, showing a cortisone-like activity [11, 12]. The benefit may depend on the synergic action of active components. The mucoadhesive properties of PVP allow the continuous release of sodium hyaluronhate, which favours re-epithelisation and wound healing, and of verbascoside, which has an anti-inflammatory action.

## Conclusions

This study showed that PHGS is relatively common in adolescents and the treatment is mainly aimed at the management of pain and eating difficulties. In Italy adolescents show up to the dentists’ observation a few days after the onset; thus, the main problem is that the lesions are already overinfected. As concerns the topical treatment, in our sample, a mouthwash containing verbascoside and sodium hyaluronhate seems to be more effective in terms of pain and resolution of the lesions than hyaluronic acid gel or chlorhexidine rinses.
